# Regional surname affinity: A spatial network approach

**DOI:** 10.1002/ajpa.23755

**Published:** 2018-12-26

**Authors:** Yongbin Shi, Le Li, Yougui Wang, Jiawei Chen, Yida Yuan, H. E. Stanley

**Affiliations:** ^1^ School of Systems Science Beijing Normal University Beijing 100875 China; ^2^ Center for Polymer Studies and Physics Department Boston University Boston MA 02215; ^3^ Institute of Genetics and Developmental Biology Chinese Academy of Sciences Beijing 100101 China

**Keywords:** community detection, ethnicity classification, isonymic distance, multilayer minimum spanning tree, spatial network

## Abstract

**Objective:**

We investigate surname affinities among areas of modern‐day China, by constructing a spatial network, and making community detection. It reports a geographical genealogy of the Chinese population that is result of population origins, historical migrations, and societal evolutions.

**Materials and methods:**

We acquire data from the census records supplied by China's National Citizen Identity Information System, including the surname and regional information of 1.28 billion registered Chinese citizens. We propose a multilayer minimum spanning tree (MMST) to construct a spatial network based on the matrix of isonymic distances, which is often used to characterize the dissimilarity of surname structure among areas. We use the fast unfolding algorithm to detect network communities.

**Results:**

We obtain a 10‐layer MMST network of 362 prefecture nodes and 3,610 edges derived from the matrix of the Euclidean distances among these areas. These prefectures are divided into eight groups in the spatial network via community detection. We measure the partition by comparing the inter‐distances and intra‐distances of the communities and obtain meaningful regional ethnicity classification.

**Discussion:**

The visualization of the resulting communities on the map indicates that the prefectures in the same community are usually geographically adjacent. The formation of this partition is influenced by geographical factors, historic migrations, trade and economic factors, as well as isolation of culture and language. The MMST algorithm proves to be effective in geo‐genealogy and ethnicity classification for it retains essential information about surname affinity and highlights the geographical consanguinity of the population.

## 1 INTRODUCTION

Over the last two decades, the use of surnames as research tools has rapidly expanded across the fields of anthropology, population geography, human population biology, and genetic genealogy (Cheshire, 2014; Colantonio, Lasker, Kaplan, & Fuster, 2003; King & Jobling, 2009; Mateos, 2007). In most countries of the world, surnames (family names) are passed down from father to son, reflect Y chromosome inheritance (Zei, Matessi, Siri, Moroni, & Cavalli‐Sforza, 1983), and thus can serve as genetic metaphor (Darlu et al., 2012). Surnames have also been applied to the study of some aspects of culture. For example, research has found that surname distributions are strongly related to language (Manni, Heeringa, & Nerbonne, 2006; Scapoli et al., 2005), migration, and social mobility (Dipierri, Ela, Rodriguez‐Larralde, & Ramallo, 2016; Longley, Webber, & Lloyd, 2007).

The most prevailing applications of surname data are geo‐genealogy and ethnicity classification that can be used by government and social scientists to define the geographical boundaries of ethnic groups. The main rationale is that isonymy is the most direct way of estimating the genetic relationship among populations from different areas (Lasker, 1977). Several empirical studies have shown that there are distinct variations in isonymy among subregions within countries, such as Argentina (Dipierri et al., 2005), Chile (Barrai et al., 2012), Paraguay (Dipierri et al., 2011), and Bolivia (Rodriguez‐Larralde et al., 2011). Du, Yuan, Hwang, Mountain, and Cavalli‐Sforza (1992) and Liu, Chen, Yuan, and Chen (2012) in particular, separate the north and south regions in China, and find that the Yangtze river and the Yellow river are the approximate boundaries. Using the isonymic distance derived from isonymy theory as input data, several clustering techniques have been proposed to regionalize surnames, including hierarchical clustering (Longley, Cheshire, & Mateos, 2011), k‐means clustering (Cheshire, Longley, & Singleton, 2010), multidimensional scaling (MDS) (Cheshire, Longley, & Mateos, 2009), self‐organizing maps (SOMs) (Rodríguez‐Díaz, Blanco‐Villegas, & Manni, 2017), and Monmonier's algorithm (Manni, Guerard, & Heyer, 2004). Many works applying these approaches into geo‐genealogy and ethnicity classification can be found in the recent literature (Mateos, 2014).

At the beginning of this century, an innovative clustering technique called “community detection” was proposed by researchers working in network science (Girvan & Newman, 2002). With the discovery of “small world” and “scale free” phenomenon (Barabási & Albert, 1999; Watts & Strogatz, 1998), complex network has attracted much attention as a new systematic way of modeling complex systems (Barabási, 2016). It can also be used to study the relationships among individuals, groups, and organizations in human societies (Borgatti, Brass, & Halgin, 2014; Szell, Lambiotte, & Thurner, 2010). Some researchers have used network approach to examine naming connections and have used the technique of community detection to identify naming communities. Mateos, Longley, and O'Sullivan (2011) took the lead when they constructed a two‐mode (bipartite) network of forename and surname associations and two one‐mode networks of surnames and forenames by using a large sample of population in 17 countries. Kowalska, Longley, and Musolesi (2015) expanded this work to 23 countries in four continents. Another approach to constructing surname networks uses similarities among surnames. Novotný and Cheshire (2012) built a Czech surname network using a similarity based on the pairwise probabilities of the co‐occurrence of surnames. By employing the well‐developed techniques of community detection to a naming network, these pioneering researchers have found that the network representation clearly defines ethno‐cultural boundaries. As far as we know, there is still little research associated with surname studies which concerns on spatial network and the community detection in it.

Chinese surnames are a significant and remarkable data source in surname studies. The cultural continuity in China is one of the oldest in the world, and its hereditary surname history dates back approximately 5,000 years (Hanks, 2003).^1^ In traditional Chinese society, the effect of the small‐scale peasant economy was such that people seldom moved from their homeland of origin (Lee, Fok, & Zhang, 2008). Families sharing the same surname tended to live together, especially in the villages (Wu, 1927), and thus the Chinese regional surname structure strongly reflects regional consanguinity and ethnicity. Chinese culture is dominated by Han culture in which the concept of patrilineal surname has been deeply rooted in the minds of people. Hence, Chinese people attach great importance to the surname and its inheritance. People cling to their surnames loyally and do not change their surnames unless some special circumstances, such as taking noble surname from the emperor, adoption. Moreover, women do not change their surnames after marriage. As a result, Chinese surnames are paternally inherited in a stable and continuous way (Yuan & Zhang, 2002). Using sampling data of surnames and a short tandem repeat on the Y‐chromosome (Y‐STR) in Shandong province, Shi et al. (2018) found that Chinese surnames can be inferred from Y‐STR profiles, indicating that Chinese surnames are an accurate data source when studying geo‐genealogy and ethnicity classification.

We here apply a community detection algorithm to a spatial network to analyze surname affinities among geographic areas and create a regional surname geography. We use a large sampling of Chinese surname data to construct the spatial networks. The network nodes are administrative regions at the prefectural level, and the edges are defined by isonymic distances. To guarantee that there are no isolated nodes in the network, we can construct it using the minimum spanning tree (MST) algorithm (Prim, 1957). Although the most essential edges are retained in the MST network, many important links are lost. To remedy this weakness, we modify this algorithm by retaining as many essential edges as possible. The new algorithm, multilayer minimum spanning tree (MMST), is an enhanced and expanded MST. We use the MMST to construct a spatial network with a topology that allows the implementation of community detection. As we will show, without any previous knowledge on the geographical information of the concerned regions, this method is able to produce a clear community structure in both topological network and geographical connections.

## 2 MATERIALS AND METHODS

### 2.1 Data and materials

Our data set from the China's National Citizen Identity Information Center (NCIIC) lists the occurrence of individual surnames in Chinese prefectures in 2007. To protect citizen privacy, the NCIIC substitutes a five‐digit number (surname ID) for each surname.^2^ Prefectures in China include prefectural level cities, autonomous prefectures, and leagues. Excluding Hong Kong, Macao, and Taiwan, the data set includes 362 prefectures. On average, there are 3.5 million people and 1,313 surnames in a prefecture.

There are 1.28 billion people listed in the data set, and they share 7,184 different surnames. Rodriguez‐Larralde et al. (2011) compared the surname data in eight European countries, three South American countries, the United States, and Yakutia, and they found the number of different surnames of most countries is more than 100,000, which is much more than that of China. China has a relatively small number of different surnames, even its population is quite large. This fact is attributed to Chinese specific surname history and culture. Chinese surname possibly originated in 5000 years ago (Hanks, 2003). At the very beginning, it was a symbol of social status and nobility, and surname included clan name and lineage name (He, Hu, Zhu, Xia, & Huang, 2016). In the Han dynasty (206 BC–220 AD), lineage names became indistinguishable from clan names, and they both evolved into modern Chinese surnames. Moreover, patrilineal inheritance of Chinese surnames has been strongly maintained and reinforced by cultural constraints. Most Chinese surnames in use today had formed for 2000 years. This has been claimed by Yuan, Zhang, Ma, and Yang (2000). They compared the distributions of 100 most common Chinese surnames in Song dynasty (960–1279 AD), Ming dynasty (1368–1644 AD), and present day, and found the three distribution curves are nearly overlapping. The stable distribution of surnames indicates that Chinese surnames are well‐preserved for a long period. However, Chinese population has risen almost twentyfold since Han dynasty. As a result, the number of different surnames of China is far less than those of most countries in the world.

China is a multiethnic country with 55 minorities. The dominant Han people comprise 91.5% of the Chinese population.^3^ Because of the differences among surname cultures, the surname structure of some minority regions differs from that of the predominantly Han regions. In particular, people in some ethnic minority groups do not use surnames, such as Yi, Miao, Tibetan, and Mongolian. Unlike most western countries, Chinese people put their surnames before first names. For the citizens without surnames whose population is very small, NCIIC took the first Chinese character of their names as their surnames. Although these surnames are not paternally inherited, they are still influenced by regional naming cultures.

### 2.2 Isonymic distance

Isonymy measures how frequent the same surnames are shared by two geographical areas (Lasker, 1977). It can also indicate the inbreeding frequency and biological relatedness within a given area (Crow, 1980; Crow & Mange, 1965; Lasker & Mascie‐Taylor, 1985). The isonymy between areas *i* and *j* can be defined as $I_{\mathrm{ij}}=\sum_{k=1}^{S}p_{\mathrm{ki}}p_{\mathrm{kj}}$, where S is the total number of surnames in both areas, *p*_*ki*_ and *p*_*kj*_ are the relative frequencies of surname *k* in the area *i* and *j*, respectively. Barrai et al. (1996) defined the inverse of isonymy in one area,$\;1/\sum_{k=1}^{S}p_{k}^{2}$, as alpha (α), which is called the effective surname number (Herrera Paz et al., 2014).

In surname studies, the isonymic distance measures the dissimilarity of surname structure between two areas. A small isonymic distance between two areas indicates that their surname structures are strongly similar. There are three ways of calculating the isonymic distance: Lasker's distance, Nei's distance and Euclidean distance. Lasker's distance is defined by *LD*_*ij*_ =−*log* (*I*_*ij*_) (Rodriguez‐Larralde et al., 1998). Nei's distance is defined by $ND_{\mathrm{ij}}=-\log\left(I_{\mathrm{ij}}/\sqrt{I_{i}I_{j}}\right)$ (Nei, 1972). Euclidean distance is defined by $ED_{\mathrm{ij}}=\sqrt{1-\sum_{k=1}^{s}\sqrt{p_{\mathrm{ki}}p_{\mathrm{kj}}}}$ (Cavalli‐Sforza & Edwards, 1967). The matrix of isonymic distances can be used to express the multilateral dissimilarities of surname structures among different areas. It is typically used as the input data in a name‐based ethnicity classification.

### 2.3 Building spatial network

A network is a collection of edges that connect nodes, and can be defined as a graph G(V, E) with a node (or vertex) set V and an edge (or link) set E. In our spatial network, the node set represents prefectures and the edge set represents interrelations between two prefectures. The original network encompasses the matrix of isonymic distances, is fully connected, and contains an enormous amount of redundant information. In contrast, an MST connects all the nodes using the minimum possible total edge weight and disallows cycles. It provides a skeletal structure with only n−1 essential edges (with relatively small isonymic distances), where n is the number of nodes in network G. Although the network constructed using the MST algorithm retains the most essential edges, it loses many important edges of the original network.

Extending the MST, we develop a MMST to build the spatial network. This algorithm aims to retain as many of important edges of the original network as possible, at the premise of removing its redundant edges. As in most schemes of ethnicity classification, the algorithm uses the matrix of isonymic distances (M) as input data. The MMST network combines L layers of MST networks, and the adjacent matrix of MMST is the sum of L adjacent matrices of MST networks. The procedure of building MMST network is an iterative process of applying the Prim's algorithm (Prim, 1957) in each step to integrate an additional MST layer derived from the remaining portion of the isonymic distance matrix. It thus guarantees that each element of the isonymic distance matrix extends to the edge of MMST at most once. When one node connects to all other nodes in the network, the iterative process of adding new layer ends, and the MMST network reaches its maximum number of layers.

### 2.4 Quantifying network dissimilarity

To investigate the change of network topology with increasing the number of layers, we calculate the network dissimilarity between two adjacent networks in the procedure of building network. We use the D‐value proposed by Schieber et al. (2017) to quantify network dissimilarity. Based on the standard information‐theoretic metrics, D‐value quantifies the differences of topological structure between networks with a three‐term function. The network dissimilarity between G and G^′^ is defined by

(1) $$D\left(G,G^{\prime }\right)=\omega_{1}\sqrt{\frac{J\left(\mu_{G},\mu_{G^{\prime }}\right)}{\log2}}+\omega_{2}\left|\sqrt{\mathrm{NND}\left(G\right)}-\sqrt{\mathrm{NN}D\left(G^{\prime }\right)}\right|+\frac{\omega_{3}}{2}\left(\sqrt{\frac{J\left(P_{\mathrm{\alpha G}},P_{\alpha G^{\prime }}\right)}{\log2}}+\sqrt{\frac{J\left(P_{\alpha G^{c}},P_{\alpha G^{c^{\prime }}}\right)}{\log2}}\right).$$

The first term on the right side of the equation focuses on the network distance distribution, μ, in which J is Jensen‐Shannon divergence. The second term characterizes the node heterogeneity, in which NND is network node dispersion of a network with diameter *d* and is given by

(2) $$\mathrm{NND}\left(G\right)=\frac{J\left(P_{1},\ldots ,P_{N}\right)}{\log d+1}.$$

The third term captures the difference of node centrality, in which *P*_*αG*_ is the alpha‐centrality distribution of network G and *G*^*c*^ is the complement of G. Here ω_1_, ω_2_, and ω_3_ are the weights of the three terms thus we have ω_1_ + *ω*_2_ + *ω*_3_ = 1.

### 2.5 Network community detection

Network communities or clusters are groups of nodes with dense internal connections. To measure the effectiveness of community detection, modularity has been proposed (Newman, 2006). High modularity levels indicate good partitioning. Modularity is defined to be the fraction of edges within the given group minus the fraction expected if edges were randomly distributed.

There are many clustering methods that can be applied to various types of networks (Fortunato, 2010). We here use the fast unfolding algorithm (Blondel, Guillaume, Lambiotte, & Lefebvre, 2008) based on an MMST network of isonymic distances to classify its nodes. This algorithm first sets initial partition and iterates it until there is no further improvement in the modularity. The modularity is a scalar value that compares the actual density of edges inside communities and the corresponding random case (Newman, 2006). This algorithm is implemented in Gephi 0.9.2,^4^ in which the resolution γ is an adjustable parameter that controls the size of community. When γ → 0, each node is a separate community. In this article, we adjust the parameter γ to get eight communities for all network community detection. By doing so, we set the parameter γ as 0.75, 0.7, and 0.6 for 9‐laye, 10‐layer, and 11‐layer MMST, respectively.

## 3 RESULTS

### 3.1 Distribution of isonymic indexes

The Supporting Information Table S1 shows the distribution of isonymic indexes in 362 Chinese prefectures. In addition to *I* and *α*, we present the number of individuals (N), the number of different surnames (S), and the ratio of number of different surnames to sample size (S/N) for prefectures in this table. The average *I* for 362 prefectures is 0.0365 ± 0.0149, which is significantly larger than those of United States (Barrai, Rodriguez‐Larralde, Mamolini, Manni, & Scapoli, 2001), France (Scapoli et al., 2005), Argentina (Dipierri et al., 2005), Honduras (Herrera Paz et al., 2014). The average *α* for 362 prefectures is 30.41 ± 9.45, which is obviously smaller than those of the countries mentioned above. S/N varies from 0.000087 (i.e., an average of 11,432 individuals per surname) to 0.017854 (i.e., an average of 56 individuals per surname) over all the prefectures. As mentioned in subsection 2.1, due to the long and stable surname inheritance, the number of modern Chinese surnames is relatively small. The number of Chinese population is so huge that the average population owned by a surname is much larger.

### 3.2 Statistical property of isonymic distances

**Figure 1:**
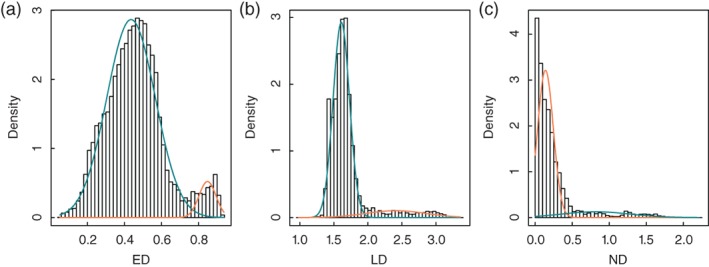
Histogram of isonymic distance with fitted densities (mixtures of normal distributions) for (a) ED, (b) LD, and (c) ND. The fitting is implemented by means of expectation conditional maximization (ECM) algorithm (see Benaglia, Chauveau, Hunter, & Young, 2009)

As mentioned above, there are three kinds of isonymic distances. We calculate these distances respectively and analyze their distribution characteristics. As shown in Figure 1, none has a normal distribution. The ED curve has two obvious peaks, and those of LD and ND have fat tails. The minor peak of the ED curve and the tails in LD and ND distributions are attributed to some minority areas where most people may have either rare surnames or no surnames, especially the prefectures in Xizang province. The averages of ED, LD, and ND for Xizang are 0.8388 ± 0.0929, 2.7504 ± 0.3232, and 1.2513 ± 0.3484, respectively. From Figure 1, we can see that almost all isonymic distances between Xizang's prefectures and other prefectures contribute to the minor peak of ED and the fat tails of LD and ND. If we remove the data of Xizang, the minor peak and fat tails of isonymic distance curves will become faint.

When building an MMST network, a good measure of isonymic distance should be more distinguishable within the range of relatively smaller values. Figure 1b,c show that LD and ND have positively skewed distributions, and that most of the values are in the small distance range. Figure 1a show that ED is a mixture of normal distributions in which the left part of the distribution is dispersed and distinguishable. As Rodriguez‐Larralde, Gonzales‐Martin, Scapoli, and Barrai (2003) argues, ED has the advantage over the other isonymic distances when few surnames are shared in two groups. Therefore, we choose the matrix of ED as input data for building the spatial network.

### 3.3 Determination of network layer

**Figure 2:**
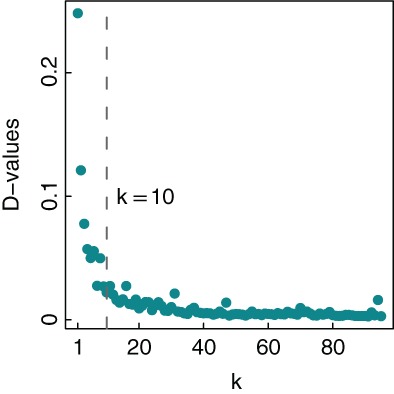
D‐values between *k*‐layer MMST and *k + 1*‐layer MMST

When we add additional layers to the original MST network, the MMST recovers some lost important edges, but an excess of additional layers can produce redundant information. An appropriate layer of network should be large enough to retain most valuable information, but not too large, and be small to minimize redundant edges. Figure 2 shows the variation of D‐value along *k* (the MMST layer), where the D‐value at *k* is calculated using the *k*‐layer MMST and the *k + 1*‐layer MMST. When the iterative process of adding layer ends, the maximum number of layers that the MMST algorithm can generate is 96. Thus *k* ∈ (1, 95). With the number of layers *k* increasing, D‐value decreases and converges to 0. This indicates that topological change of the spatial network decreases as *k* increases, and that eventually ceases when the scale of network becomes sufficiently large. The rationale of determining layer is to choose a turning point that D‐value falls rapidly before it and remains nearly unchanged after it. Figure 2 shows that the D‐value around 10 matches this criteria. We thus select 10 as the number of MMST layer. In fact, networks with the numbers of layer around 10 have topological structures similar to that of 10‐layer MMST, as well as their clustering results (The clustering results based on 9‐layer MMST and 11‐layer MMST are shown in the Supporting Information Figure S1 and S2, respectively).

### 3.4 Spatial network

**Figure 3:**
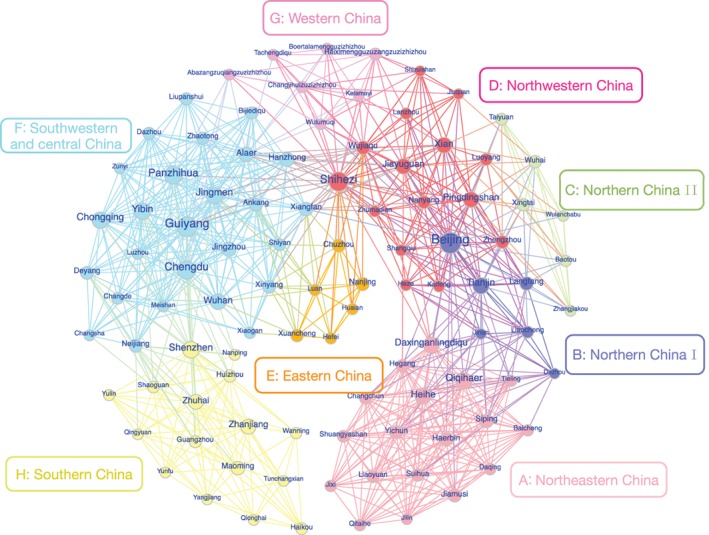
Visualization of the constructed spatial network. To better illustrate the network, we exclude the nodes whose degree is less than 24 and thus only 86 nodes and 663 links of the network are remained in this figure. The size of cycles (refer to nodes) is directly proportional to the degree of nodes, and the thickness of lines is inversely proportional to the weight (ED) of links. The colors of nodes represent the results of community detection

Figure 3 shows a spatial network generated using the Fruchterman–Reingold algorithm (Fruchterman & Reingold, 1991) and visualized using Gephi 0.9.2. This spatial network contains 362 nodes and 3,610 edges. On average, there are approximately 20 edges connected to one node. The minimum degree and maximum degree are 10 and 71, respectively. The degree distribution exhibits a very skewed character that can be fitted as a power‐law form with an exponent of 4.22.^5^ As we can see from Figure 3, the sizes of several nodes (which are prefectures) are obviously bigger than those of the rest. These prefectures are usually regional centers of social, economic and cultural activities, such as Beijing, Guiyang, and Chengdu. Some of them are transportation hubs, such as Jiayuguan, Qiqihaer, and Chongqing. They can also be emerging immigrant cities, in which immigrants come from multiple sources, such as Shihezi, Shenzhen, and Panzhihua.

We detect communities in the spatial network by using the fast unfolding algorithm. The prefectures are grouped into eight communities. According to the corresponding geographical locations in China, these communities are marked using letters A through H, and labeled by Northeastern China, Northern China I, Northern China II, Northwestern China, Eastern China, Southwestern & Central China, Western China, and Southern China respectively.

**Figure 4:**
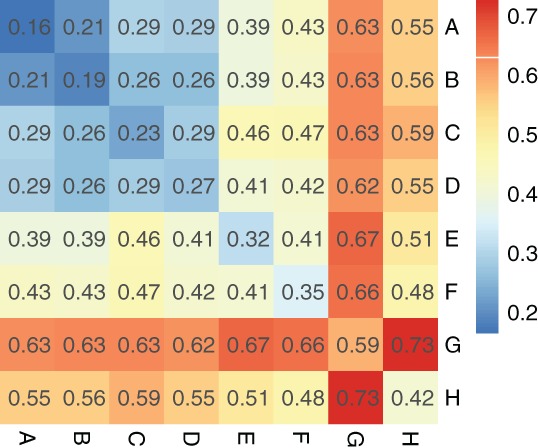
Heat map of the matrix of AED. The rows (or columns) correspond to communities. Each cell is colorized based on its corresponding value of AED

To determine the accuracy of the community detection based on the network topology, we calculate the average ED (denoted by AED) between any two communities and within a community. The former is the average of Euclidean distances between all possible two‐community prefecture pairs, and the latter is the average of all Euclidean distances between any two prefectures within the corresponding community. Figure 4 shows AED matrix as a heat map in which its elements are the corresponding AEDs. With the one exception of the AED between communities B and D, all inter‐group distances are larger than the corresponding intra‐group distances. It indicates that the prefectures within a community have similar surname structure with each other rather than inter‐community. Although the edges of the resulting spatial network are limited, it exhibits a strong community partition. Note that the AEDs between and within A–D communities, including the exceptional AED, are smaller than 0.3, and almost all prefectures of A–D communities locate in northern areas of China. This is in accord with the study of Yuan and Zhang (2002) that the flat landscape of northern China has made the population more mobile than those in southern China. However, we can distinguish A–D communities by detecting community in the spatial network even though they are much similar with each other on surname structure.

## 4 DISCUSSION

The community structure in the MMST, as shown in Figure 3, is obtained by computing the similarity of surname structure between prefectures. Only topological properties of the network are taken into account rather than geographical ones in this procedure. The community partition is evaluated by comparing the inter‐community distances with intra‐community ones and the results are presented in Figure 4. To justify the efficacy of this partition, we also need to evaluate the closeness of the prefectures within each community shown in Figure 3 from a geographical viewpoint.

**Figure 5:**
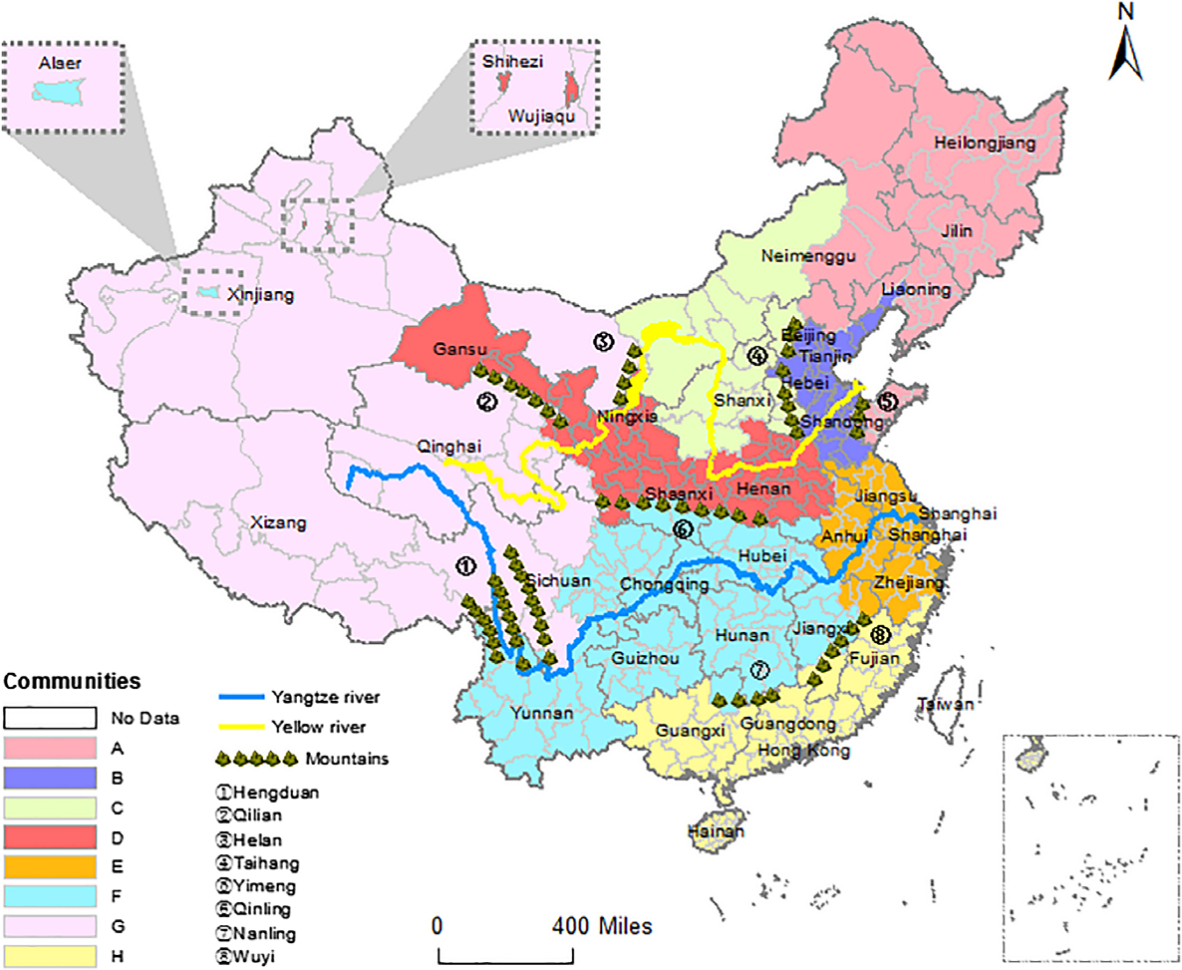
Map showing the allocations of eight communities in the spatial network. The map was developed by ArcGis 10.2.2

Figure 5 shows a map of eight communities in the spatial network. Note that the regions in one community are usually geographically adjacent, reflecting the Tobler's First Law of Geography: everything is related to everything else, but nearby things are more closely related than distant things (Tobler, 1970). Note also that several provinces are separated into different communities, indicating that some prefectures of one province are more similar to certain prefectures of other provinces, and that this resulting ethnicity classification is not coincident to administrative divisions.

Some geographical factors affect the community detection results. Waterways promote human mobility, and mountains hinder it. As shown in Figure 5, the Yangtze river cuts through three communities, while the Yellow river cuts through four communities, and enters community D twice. The Yangtze river and the Yellow river are the two longest rivers in China, and both pass China through west to east. The difference between them is, the former is a waterway channel and characterized by a large water discharge and a deep‐incised valley, the latter is not an efficient way of transportation and characterized by huge sediment discharge and steep longitudinal profile (Saito, Yang, & Hori, 2001). In contrast to the Yellow river, the Yangtze river is much more convenient for human mobility, the upstream and downstream of the Yangtze river are more likely to be grouped into the same community. Especially, community F is located along the Yangtze river, and wraps a large part of the river. We can also find that the Hengduan Mountains define the geographical boundary between G and F communities. Similar blocking effect can be found in the boundaries between different communities, which are defined respectively by Qilian Mountains (between D and G), Helan Mountains (between C and D, G), Taihang Mountians (between B and C), Yimeng Mountains (between A and B in Shandong province), Qinling Mountains (between D and F), Nanling Mountains and Wuyi Mountains (between F and H).

Historic migration can also effect separations within provinces. The division of the prefectures within Shandong province reflects a historic migration from Shandong to the three northeastern provinces (Heilongjiang, Jilin, and Liaoning), which is the well‐known “Rush to Northeast”. This mass migration had lasted for more than 300 years from the early Qing dynasty to the end of the last century. Natural disasters and excessive population density prompted the citizens of Shandong to leave their homeland and make their living elsewhere. Because some prefectures in Shandong border the Bohai sea, some emigrants there used the seaway to move to northeastern provinces. Those in prefectures not bordering the sea used land routes for their migrations. These two transportation methods split Shandong province into two parts with different surname structures. Note that the north part of Shaanxi province and most of Shanxi province share the community C with the eastern part of Neimenggu, reflecting the well‐known “Going to the West Gate” migration. This historic migration had lasted for approximately 400 years, from the middle of Ming dynasty to the early Chinese Republic. People in Shaanxi and Shanxi moved to Neimenggu because of its commercial viability, fertile land, and peaceful environment.

From the perspective of migration, community F is the result of the consecutive mass migrations of “Jiangxi Fills Huguang” and “Huguang Fills Sichuan”, where “Huguang” refers to the areas of modern Hunan province and Hubei province. Especially during Yuan‐Ming and Ming‐Qing transitional periods, due to the political and economic distresses a lot of people migrated from Jiangxi to Huguang, and from Huguang to Sichuan. We trace the routes of the three historical migrations mentioned above on a map of China in the Supporting Information Figure S3.

There are also outliers geographically distant from the center of their communities. Three typical cases, Alaer, Shihezi, and Wujiaqu, are actually the prescribed locations of the Production and Construction Corps (PCC) in Xinjiang province. PCC was established in 1954 to reclaim and cultivate soil and to consolidate the border areas (McMillen, 1981). The massive population influxes shaped the composition of these three prefectures and caused their surname structure to differ from those of nearby prefectures (Liu, 2007). These three outlier prefectures are small but appear as high‐degree nodes in the network graph (see Figure 3) because the surname structure here is similar to the former prefectures of the immigrants. We also find that Shihezi and Wujiaqu belong to the community D, while Alaer belongs to the community F. This fact indicates that most migrants of Shihezi and Wujiaqu were from the north, while those of Alaer were from the south.

In addition to geographical factors and historic migrations we mentioned above, there are many other factors contribute to the result of population divisions, such as trade and economic factors, as well as isolation of culture and language. The western part of community D is the famous “Hexi Corridor”. As a part of the “Northern Silk Road”, it had been the most important route from north China to central Asia for traders, and also promoted population integration along the route. The formation of community E is also partly caused by trade and economic factors. Community E is the region encircling Yangtze Delta economic zone, which embodies some historical or modern economically developed prefectures, such as Suzhou, Nanking, and Shanghai. The former two cities show the two greatest longevity (2,430 and 2158 years, respectively) through their being the largest 50 cities in world from 430 BC to AD 2000 (Batty, 2006), and the latter one is the commercial and financial center of China nowadays. A long regional economic development has promoted population mobility within Yangtze Delta economic zone. The formation of community G and H is typically caused by isolation of culture and language. They are located respectively in the most western and southeastern of China. The people live in both areas have formed their own regional cultures and languages that are quite different from those elsewhere.

In fact, the factors we mentioned above sometimes take their effects together. For example, “Hexi Corridor” is an important route of trade, which was determined by its geographical condition. There are many oases along the path, and it borders Qilian Mountains to the south, Gobi desert to the north. Some historical migrations were also partly driven by economic factors. A typical case is “Going to the West Gate”.

In contrast to the clustering result derived from k‐means clustering (see Figure S4 in Supporting Information), the eight community allocations derived from MMST network (see Figure 5) are not only clear and intuitive but also sensible and reasonable. In the k‐means clustering algorithm, prefectures with small isonymic distance are grouped into one cluster. However, in our approach, prefectures are in a community only if they are densely connected with one another in the MMST network. In the MMST network, all prefectures only have several most relevant connections. Two prefectures are grouped into one cluster by k‐means and may be far away from each other in our network topology. The prefectures of some clusters scatter over the map in Supporting Information Figure S4, while those in Figure 5 are basically continuous and complete. The western China is a densely populated area of minor ethnic groups in which even some people do not have inheritable surname, and thus its surname structure differs from those in other places. As shown in Figure 5, the prefectures in the western China are mainly in the community G, while they are partitioned into several groups in Supporting Information Figure S4.

In summary, the construction of MMST network combining with the community detection in it is an effective approach to study regional surname affinities. This algorithm guarantees the connectivity of network, in which no nodes are isolated. It also ensures that all nodes have at least L (the number of layers in MMST) strongest links of them. The viewpoint of network topology is the most essential merit of MMST that makes this algorithm different from traditional clustering techniques. In other words, the traditional clustering techniques are prone to offer biased results of ethnicity classification, for the reason that they are equivalent to community detection in fully connected weighted network, which overemphasizes surname kinship and downgrades geographical consanguinity. In contrast, the topology of the MMST spatial network yields meaningful results of geo‐genealogy and ethnicity classification. The MMST algorithm can also be used to filter relevant information in many other issues. Our work here calls for a deep mining of the spatial and surname networks to reveal more hidden patterns in the surname data set.

## Supporting information

Appendix S1 Supplementary Material

## Footnotes

1 By contrast, hereditary surnames of other countries and areas formed latterly, such as European surnames originated in the first century BC, Japanese and Korean surnames originated in the fifth century AD.

2 The five‐digit numbers are assigned in the order of surnames that occur in the data processing, for example, 10,001 is assigned to the first surname, 10,002 is the second, and so on.

3 See official figures reported in the National Bureau of Statistics of China (www.stats.gov.cn).

4 It is available at https://gephi.org/.

5 The fitting was performed by using Newman's algorithm (see Clauset, Shalizi, & Newman, 2009).
