# Supplementary material for: Regional surname affinity: A spatial network approach
Source: Am J Phys Anthropol. 2018 Dec 26;168(3):428–37. doi: 10.1002/ajpa.23755 (PMC6590414; doi:10.1002/ajpa.23755)
Supplement: Supplementary file 1 — Appendix S1 Supplementary Material [file AJPA-168-428-s001.docx]

Table S1 – Prefecture of China; number of individuals, N; number of different surnames, S; isonymy, I; effective surname number, $\alpha$; ratio of number of different surnames to sample size, S/N.

| Prefecture | N | S | I | $\alpha$ | S/N |
| --- | --- | --- | --- | --- | --- |
| Beijing | 11885924 | 1941 | 0.0379 | 26.40 | 0.000163 |
| Tianjin | 9484351 | 1600 | 0.0434 | 23.06 | 0.000169 |
| Shijiazhuang | 9338194 | 1832 | 0.0390 | 25.62 | 0.000196 |
| Tangshan | 7166925 | 1428 | 0.0441 | 22.67 | 0.000199 |
| Qinhuangdao | 2762176 | 1231 | 0.0416 | 24.06 | 0.000446 |
| Handan | 8698251 | 1923 | 0.0415 | 24.08 | 0.000221 |
| Xingtai | 6689962 | 1711 | 0.0401 | 24.97 | 0.000256 |
| Baoding | 10763245 | 2195 | 0.0442 | 22.62 | 0.000204 |
| Zhangjiakou | 4521991 | 1398 | 0.0389 | 25.71 | 0.000309 |
| Chengde | 3583587 | 1258 | 0.0399 | 25.03 | 0.000351 |
| Cangzhou | 6793341 | 1786 | 0.0441 | 22.69 | 0.000263 |
| Langfang | 3916712 | 1506 | 0.0447 | 22.35 | 0.000385 |
| Hengshui | 4157682 | 1372 | 0.0441 | 22.66 | 0.000330 |
| Taiyuan | 3364830 | 1359 | 0.0386 | 25.94 | 0.000404 |
| Datong | 3007255 | 1194 | 0.0384 | 26.06 | 0.000397 |
| Yangquan | 1260699 | 867 | 0.0457 | 21.88 | 0.000688 |
| Changzhi | 3177500 | 1579 | 0.0433 | 23.08 | 0.000497 |
| Jincheng | 2109454 | 960 | 0.0417 | 23.99 | 0.000455 |
| Shuozhou | 1467033 | 1233 | 0.0369 | 27.12 | 0.000840 |
| Jinzhong | 3088520 | 1331 | 0.0382 | 26.17 | 0.000431 |
| Yuncheng | 4896580 | 1789 | 0.0366 | 27.35 | 0.000365 |
| Xinzhou | 2935261 | 1439 | 0.0412 | 24.30 | 0.000490 |
| Linfen | 4103140 | 1917 | 0.0375 | 26.69 | 0.000467 |
| Luliang | 3575104 | 1480 | 0.0428 | 23.37 | 0.000414 |
| Huhehaote | 2126121 | 1815 | 0.0368 | 27.19 | 0.000854 |
| Baotou | 2097550 | 1650 | 0.0388 | 25.77 | 0.000787 |
| Wuhai | 430025 | 908 | 0.0391 | 25.59 | 0.002112 |
| Chifeng | 4470779 | 2037 | 0.0365 | 27.41 | 0.000456 |
| Tongliao | 3087979 | 2176 | 0.0306 | 32.69 | 0.000705 |
| Eerduosi | 1396485 | 1516 | 0.0367 | 27.23 | 0.001086 |
| Hulunbeier | 2676628 | 1886 | 0.0332 | 30.12 | 0.000705 |
| Bayannaoer | 1756491 | 1631 | 0.0404 | 24.77 | 0.000929 |
| Wulanchabu | 2728115 | 1729 | 0.0379 | 26.37 | 0.000634 |
| Xinganmeng | 1628813 | 1708 | 0.0300 | 33.35 | 0.001049 |
| Xilinguolemeng | 943177 | 1595 | 0.0236 | 42.40 | 0.001691 |
| Alashanmeng | 181032 | 1075 | 0.0269 | 37.16 | 0.005938 |
| Shenyang | 7000398 | 1460 | 0.0355 | 28.18 | 0.000209 |
| Dalian | 5649036 | 1254 | 0.0358 | 27.96 | 0.000222 |
| Anshan | 3484033 | 1102 | 0.0364 | 27.49 | 0.000316 |
| Fushun | 2239649 | 1094 | 0.0354 | 28.28 | 0.000488 |
| Benxi | 1563243 | 927 | 0.0334 | 29.92 | 0.000593 |
| Dandong | 2427088 | 932 | 0.0323 | 30.97 | 0.000384 |
| Jinzhou | 3086053 | 1206 | 0.0406 | 24.62 | 0.000391 |
| Yingkou | 2297040 | 1040 | 0.0355 | 28.15 | 0.000453 |
| Fuxin | 1924335 | 1148 | 0.0369 | 27.10 | 0.000597 |
| Liaoyang | 1823990 | 941 | 0.0365 | 27.38 | 0.000516 |
| Panjin | 1258515 | 1052 | 0.0413 | 24.22 | 0.000836 |
| Tieling | 3033169 | 1208 | 0.0383 | 26.13 | 0.000398 |
| Chaoyang | 3387572 | 1039 | 0.0408 | 24.48 | 0.000307 |
| Huludao | 2741603 | 1167 | 0.0433 | 23.07 | 0.000426 |
| Changchun | 7311905 | 1625 | 0.0400 | 25.01 | 0.000222 |
| Jilin | 4278921 | 1339 | 0.0365 | 27.42 | 0.000313 |
| Siping | 3273365 | 1579 | 0.0407 | 24.55 | 0.000482 |
| Liaoyuan | 1226724 | 1021 | 0.0367 | 27.25 | 0.000832 |
| Tonghua | 2263476 | 1237 | 0.0353 | 28.34 | 0.000547 |
| Baishan | 1289388 | 980 | 0.0372 | 26.91 | 0.000760 |
| Songyuan | 2748532 | 1412 | 0.0392 | 25.52 | 0.000514 |
| Baicheng | 2013790 | 1233 | 0.0395 | 25.35 | 0.000612 |
| Yanbianchaoxianzuzizhizhou | 2166847 | 1089 | 0.0363 | 27.56 | 0.000503 |
| Haerbin | 9685934 | 1834 | 0.0363 | 27.58 | 0.000189 |
| Qiqihaer | 5580348 | 1520 | 0.0382 | 26.18 | 0.000272 |
| Jixi | 1891465 | 1106 | 0.0353 | 28.30 | 0.000585 |
| Hegang | 1076129 | 954 | 0.0363 | 27.53 | 0.000887 |
| Shuangyashan | 1493350 | 1013 | 0.0373 | 26.85 | 0.000678 |
| Daqing | 2677404 | 1246 | 0.0381 | 26.24 | 0.000465 |
| Yichun | 1258810 | 1013 | 0.0377 | 26.55 | 0.000805 |
| Jiamusi | 2447474 | 1327 | 0.0364 | 27.49 | 0.000542 |
| Qitaihe | 823196 | 875 | 0.0362 | 27.60 | 0.001063 |
| Mudanjiang | 2643287 | 1164 | 0.0337 | 29.69 | 0.000440 |
| Heihe | 1697962 | 1152 | 0.0373 | 26.83 | 0.000678 |
| Suihua | 5664417 | 1549 | 0.0393 | 25.42 | 0.000273 |
| Daxinganlingdiqu | 509481 | 898 | 0.0380 | 26.32 | 0.001763 |
| Shanghai | 13681600 | 1559 | 0.0228 | 43.95 | 0.000114 |
| Nanjing | 5950461 | 1495 | 0.0246 | 40.73 | 0.000251 |
| Wuxi | 4519827 | 1092 | 0.0211 | 47.44 | 0.000242 |
| Xuzhou | 9073163 | 1935 | 0.0343 | 29.16 | 0.000213 |
| Changzhou | 3510354 | 1094 | 0.0218 | 45.93 | 0.000312 |
| Suzhou | 6093242 | 1288 | 0.0240 | 41.67 | 0.000211 |
| Nantong | 7699450 | 1259 | 0.0241 | 41.42 | 0.000164 |
| Lianyungang | 4759429 | 1210 | 0.0314 | 31.80 | 0.000254 |
| Huaian | 5229716 | 1449 | 0.0267 | 37.41 | 0.000277 |
| Yancheng | 7923093 | 1313 | 0.0276 | 36.19 | 0.000166 |
| Yangzhou | 4565804 | 1195 | 0.0243 | 41.07 | 0.000262 |
| Zhenjiang | 2655525 | 1098 | 0.0230 | 43.56 | 0.000413 |
| Taizhou | 5012160 | 1410 | 0.0241 | 41.56 | 0.000281 |
| Suqian | 5194972 | 1415 | 0.0300 | 33.39 | 0.000272 |
| Hangzhou | 6600160 | 1407 | 0.0196 | 51.11 | 0.000213 |
| Ningbo | 5569592 | 1200 | 0.0228 | 43.78 | 0.000215 |
| Wenzhou | 7410846 | 1358 | 0.0343 | 29.17 | 0.000183 |
| Jiaxing | 3342074 | 982 | 0.0258 | 38.72 | 0.000294 |
| Huzhou | 2576678 | 1099 | 0.0231 | 43.38 | 0.000427 |
| Shaoxing | 4347525 | 987 | 0.0230 | 43.48 | 0.000227 |
| Jinhua | 4572491 | 1005 | 0.0252 | 39.73 | 0.000220 |
| Quzhou | 2451803 | 1036 | 0.0279 | 35.80 | 0.000423 |
| Zhoushan | 950853 | 645 | 0.0241 | 41.47 | 0.000678 |
| Taizhou | 5594395 | 1167 | 0.0324 | 30.88 | 0.000209 |
| Lishui | 2521621 | 829 | 0.0280 | 35.67 | 0.000329 |
| Hefei | 4605410 | 1712 | 0.0262 | 38.24 | 0.000372 |
| Wuhu | 2270305 | 1216 | 0.0219 | 45.66 | 0.000536 |
| Bengbu | 3499699 | 1538 | 0.0304 | 32.89 | 0.000439 |
| Huainan | 2356568 | 1515 | 0.0301 | 33.17 | 0.000643 |
| Maanshan | 1251558 | 1037 | 0.0237 | 42.27 | 0.000829 |
| Huaibei | 2108839 | 1494 | 0.0368 | 27.21 | 0.000708 |
| Tongling | 722880 | 821 | 0.0226 | 44.34 | 0.001136 |
| Anqing | 6045560 | 1231 | 0.0251 | 39.91 | 0.000204 |
| Huangshan | 1472900 | 776 | 0.0360 | 27.76 | 0.000527 |
| Chuzhou | 4356596 | 1562 | 0.0273 | 36.63 | 0.000359 |
| Fuyang | 9338494 | 3080 | 0.0373 | 26.77 | 0.000330 |
| Suzhou | 6022946 | 1999 | 0.0360 | 27.78 | 0.000332 |
| Chaohu | 4471348 | 1405 | 0.0232 | 43.19 | 0.000314 |
| Luan | 6829111 | 1812 | 0.0274 | 36.51 | 0.000265 |
| Bozhou | 5498339 | 3185 | 0.0341 | 29.35 | 0.000579 |
| Chizhou | 1567222 | 847 | 0.0244 | 41.05 | 0.000540 |
| Xuancheng | 2734296 | 1224 | 0.0224 | 44.72 | 0.000448 |
| Fuzhou | 6033862 | 1274 | 0.0653 | 15.32 | 0.000211 |
| Xiamen | 1562357 | 891 | 0.0487 | 20.53 | 0.000570 |
| Putian | 3012955 | 853 | 0.0627 | 15.94 | 0.000283 |
| Sanming | 2655773 | 874 | 0.0331 | 30.22 | 0.000329 |
| Quanzhou | 6598683 | 1098 | 0.0476 | 21.00 | 0.000166 |
| Zhangzhou | 4692702 | 797 | 0.0494 | 20.22 | 0.000170 |
| Nanping | 3033053 | 984 | 0.0290 | 34.53 | 0.000324 |
| Longyan | 2849657 | 782 | 0.0319 | 31.32 | 0.000274 |
| Ningde | 3228936 | 972 | 0.0388 | 25.76 | 0.000301 |
| Nanchang | 4752792 | 1493 | 0.0243 | 41.11 | 0.000314 |
| Jingdezhen | 1515748 | 879 | 0.0259 | 38.63 | 0.000580 |
| Pingxiang | 1791521 | 725 | 0.0317 | 31.59 | 0.000405 |
| Jiujiang | 4657585 | 1411 | 0.0206 | 48.52 | 0.000303 |
| Xinyu | 1114843 | 727 | 0.0289 | 34.65 | 0.000652 |
| Yingtan | 1106509 | 787 | 0.0236 | 42.41 | 0.000711 |
| Ganzhou | 8466335 | 1157 | 0.0332 | 30.09 | 0.000137 |
| Jian | 4654351 | 965 | 0.0348 | 28.75 | 0.000207 |
| Yichun | 5117448 | 1025 | 0.0225 | 44.43 | 0.000200 |
| Fuzhou | 3766869 | 1132 | 0.0242 | 41.31 | 0.000301 |
| Shangrao | 6810172 | 1494 | 0.0237 | 42.13 | 0.000219 |
| Jinan | 5990752 | 1501 | 0.0425 | 23.53 | 0.000251 |
| Qingdao | 7430590 | 1336 | 0.0380 | 26.35 | 0.000180 |
| Zibo | 4161915 | 1062 | 0.0382 | 26.15 | 0.000255 |
| Zaozhuang | 3795325 | 1263 | 0.0399 | 25.08 | 0.000333 |
| Dongying | 1805929 | 1001 | 0.0426 | 23.47 | 0.000554 |
| Yantai | 6458757 | 1230 | 0.0384 | 26.07 | 0.000190 |
| Weifang | 8554315 | 1379 | 0.0472 | 21.19 | 0.000161 |
| Jining | 8113203 | 1505 | 0.0366 | 27.30 | 0.000186 |
| Taian | 5535801 | 1217 | 0.0396 | 25.23 | 0.000220 |
| Weihai | 2499416 | 932 | 0.0391 | 25.55 | 0.000373 |
| Rizhao | 2815079 | 1606 | 0.0325 | 30.76 | 0.000570 |
| Laiwu | 1245856 | 701 | 0.0397 | 25.18 | 0.000563 |
| Linyi | 10210380 | 2042 | 0.0397 | 25.17 | 0.000200 |
| Dezhou | 5551761 | 1483 | 0.0445 | 22.45 | 0.000267 |
| Liaocheng | 5725814 | 1657 | 0.0377 | 26.50 | 0.000289 |
| Binzhou | 3748342 | 1095 | 0.0473 | 21.16 | 0.000292 |
| Heze | 8985419 | 1885 | 0.0376 | 26.61 | 0.000210 |
| Zhengzhou | 6837333 | 1652 | 0.0401 | 24.92 | 0.000242 |
| Kaifeng | 4917637 | 1713 | 0.0383 | 26.14 | 0.000348 |
| Luoyang | 6552059 | 1705 | 0.0380 | 26.31 | 0.000260 |
| Pingdingshan | 5038756 | 1678 | 0.0406 | 24.62 | 0.000333 |
| Anyang | 5498035 | 1539 | 0.0418 | 23.90 | 0.000280 |
| Hebi | 1486677 | 968 | 0.0451 | 22.19 | 0.000651 |
| Xinxiang | 5708933 | 1862 | 0.0403 | 24.84 | 0.000326 |
| Jiaozuo | 3535304 | 1280 | 0.0370 | 27.03 | 0.000362 |
| Puyang | 3750356 | 1529 | 0.0382 | 26.17 | 0.000408 |
| Xuchang | 4616324 | 1549 | 0.0372 | 26.90 | 0.000336 |
| Luohe | 2640220 | 1269 | 0.0373 | 26.83 | 0.000481 |
| Sanmenxia | 2236055 | 1203 | 0.0402 | 24.87 | 0.000538 |
| Nanyang | 10878753 | 2080 | 0.0380 | 26.32 | 0.000191 |
| Shangqiu | 8735325 | 2055 | 0.0378 | 26.46 | 0.000235 |
| Xinyang | 8130267 | 2059 | 0.0282 | 35.51 | 0.000253 |
| Zhoukou | 11219568 | 2569 | 0.0413 | 24.21 | 0.000229 |
| Zhumadian | 8508062 | 1942 | 0.0374 | 26.70 | 0.000228 |
| Jiyuan | 675139 | 680 | 0.0508 | 19.69 | 0.001007 |
| Wuhan | 7893845 | 1802 | 0.0246 | 40.67 | 0.000228 |
| Huangshi | 2512622 | 1081 | 0.0258 | 38.77 | 0.000430 |
| Shiyan | 3444443 | 1327 | 0.0277 | 36.12 | 0.000385 |
| Yichang | 3959305 | 1395 | 0.0253 | 39.60 | 0.000352 |
| Xiangfan | 5600505 | 2376 | 0.0305 | 32.79 | 0.000424 |
| Ezhou | 1038566 | 759 | 0.0240 | 41.60 | 0.000731 |
| Jingmen | 2918530 | 1396 | 0.0287 | 34.86 | 0.000478 |
| Xiaogan | 5080062 | 1620 | 0.0256 | 39.05 | 0.000319 |
| Jingzhou | 6404010 | 1870 | 0.0269 | 37.19 | 0.000292 |
| Huanggang | 7209411 | 1788 | 0.0237 | 42.18 | 0.000248 |
| Xianning | 2758165 | 1189 | 0.0230 | 43.44 | 0.000431 |
| Suizhou | 2523438 | 2123 | 0.0260 | 38.48 | 0.000841 |
| Enshitujiazumiaozuzizhizhou | 3851014 | 1281 | 0.0259 | 38.58 | 0.000333 |
| Xiantao | 1464461 | 1036 | 0.0256 | 39.09 | 0.000707 |
| Qianjiang | 985913 | 866 | 0.0265 | 37.68 | 0.000878 |
| Tianmen | 1717714 | 1143 | 0.0269 | 37.15 | 0.000665 |
| Shennongjialinqu | 79066 | 369 | 0.0279 | 35.84 | 0.004667 |
| Changsha | 6264830 | 1450 | 0.0267 | 37.49 | 0.000231 |
| Zhuzhou | 3772516 | 1055 | 0.0289 | 34.62 | 0.000280 |
| Xiangtan | 2876282 | 906 | 0.0296 | 33.77 | 0.000315 |
| Hengyang | 7384048 | 1423 | 0.0310 | 32.26 | 0.000193 |
| Shaoyang | 7429726 | 1248 | 0.0304 | 32.93 | 0.000168 |
| Yueyang | 5339803 | 1345 | 0.0269 | 37.22 | 0.000252 |
| Changde | 6100648 | 1361 | 0.0232 | 43.09 | 0.000223 |
| Zhangjiajie | 1608881 | 836 | 0.0263 | 38.05 | 0.000520 |
| Yiyang | 4540621 | 1350 | 0.0266 | 37.65 | 0.000297 |
| Chenzhou | 4657057 | 1142 | 0.0402 | 24.86 | 0.000245 |
| Yongzhou | 5767342 | 1500 | 0.0324 | 30.82 | 0.000260 |
| Huaihua | 4935022 | 1316 | 0.0313 | 31.90 | 0.000267 |
| Loudi | 4213184 | 981 | 0.0341 | 29.36 | 0.000233 |
| Xiangxitujiazumiaozuzizhizhou | 2717122 | 955 | 0.0381 | 26.24 | 0.000351 |
| Guangzhou | 7277496 | 1308 | 0.0269 | 37.23 | 0.000180 |
| Shaoguan | 3169439 | 1070 | 0.0285 | 35.14 | 0.000338 |
| Shenzhen | 1905297 | 1186 | 0.0274 | 36.48 | 0.000622 |
| Zhuhai | 910447 | 854 | 0.0324 | 30.90 | 0.000938 |
| Shantou | 4861067 | 810 | 0.0552 | 18.13 | 0.000167 |
| Foshan | 3551386 | 881 | 0.0303 | 32.99 | 0.000248 |
| Jiangmen | 3868520 | 890 | 0.0369 | 27.11 | 0.000230 |
| Zhanjiang | 7285762 | 1608 | 0.0417 | 23.97 | 0.000221 |
| Maoming | 6896027 | 1461 | 0.0349 | 28.66 | 0.000212 |
| Zhaoqing | 4006872 | 984 | 0.0315 | 31.70 | 0.000246 |
| Huizhou | 3029013 | 1055 | 0.0325 | 30.72 | 0.000348 |
| Meizhou | 5086566 | 892 | 0.0364 | 27.49 | 0.000175 |
| Shanwei | 3146220 | 959 | 0.0442 | 22.64 | 0.000305 |
| Heyuan | 3355395 | 950 | 0.0369 | 27.12 | 0.000283 |
| Yangjiang | 2639007 | 1091 | 0.0352 | 28.41 | 0.000413 |
| Qingyuan | 3940741 | 1034 | 0.0307 | 32.60 | 0.000262 |
| Dongguan | 1629733 | 795 | 0.0340 | 29.44 | 0.000488 |
| Zhongshan | 1382220 | 643 | 0.0426 | 23.49 | 0.000465 |
| Chaozhou | 2518980 | 598 | 0.0504 | 19.82 | 0.000237 |
| Jieyang | 5976196 | 1166 | 0.0517 | 19.35 | 0.000195 |
| Yunfu | 2646651 | 765 | 0.0386 | 25.93 | 0.000289 |
| Nanning | 6617134 | 1419 | 0.0384 | 26.03 | 0.000214 |
| Liuzhou | 3565920 | 1242 | 0.0373 | 26.78 | 0.000348 |
| Guilin | 4951741 | 1232 | 0.0268 | 37.30 | 0.000249 |
| Wuzhou | 3066716 | 738 | 0.0355 | 28.14 | 0.000241 |
| Beihai | 1500285 | 836 | 0.0343 | 29.19 | 0.000557 |
| Fangchenggang | 800815 | 629 | 0.0412 | 24.26 | 0.000785 |
| Qinzhou | 3430916 | 959 | 0.0386 | 25.91 | 0.000280 |
| Guigang | 4758258 | 1060 | 0.0360 | 27.79 | 0.000223 |
| Yulin | 5959486 | 1381 | 0.0348 | 28.75 | 0.000232 |
| Baise | 3746901 | 1219 | 0.0683 | 14.63 | 0.000325 |
| Hezhou | 2121097 | 964 | 0.0260 | 38.40 | 0.000454 |
| Hechi | 3856103 | 1201 | 0.0735 | 13.61 | 0.000311 |
| Laibin | 2474415 | 798 | 0.0474 | 21.09 | 0.000323 |
| Chongzuo | 2313411 | 956 | 0.0602 | 16.60 | 0.000413 |
| Haikou | 1475562 | 1137 | 0.0479 | 20.88 | 0.000771 |
| Sanya | 562281 | 705 | 0.0361 | 27.67 | 0.001254 |
| Wuzhishan | 112551 | 426 | 0.1115 | 8.97 | 0.003785 |
| Qionghai | 468253 | 476 | 0.0477 | 20.96 | 0.001017 |
| Danzhou | 939540 | 815 | 0.0453 | 22.10 | 0.000867 |
| Wenchang | 546552 | 445 | 0.0559 | 17.89 | 0.000814 |
| Wanning | 575000 | 559 | 0.0448 | 22.34 | 0.000972 |
| Dongfang | 400746 | 522 | 0.0572 | 17.49 | 0.001303 |
| Dinganxian | 319532 | 409 | 0.0583 | 17.17 | 0.001280 |
| Tunchangxian | 128461 | 385 | 0.0567 | 17.64 | 0.002997 |
| Chengmaixian | 478914 | 702 | 0.0911 | 10.98 | 0.001466 |
| Lingaoxian | 435955 | 428 | 0.1004 | 9.96 | 0.000982 |
| Baishalizuzizhixian | 185827 | 440 | 0.0753 | 13.28 | 0.002368 |
| Changjianglizuzizhixian | 241698 | 453 | 0.0404 | 24.76 | 0.001874 |
| Ledonglizuzizhixian | 490862 | 553 | 0.0505 | 19.78 | 0.001127 |
| Lingshuilizuzizhixian | 346280 | 417 | 0.0511 | 19.55 | 0.001204 |
| Baotinglizumiaozuzizhixian | 162432 | 477 | 0.0738 | 13.55 | 0.002937 |
| Qiongzhonglizumiaozuzizhixian | 211975 | 504 | 0.1015 | 9.85 | 0.002378 |
| Chongqing | 30968906 | 2709 | 0.0248 | 40.29 | 0.000087 |
| Chengdu | 10878513 | 2037 | 0.0262 | 38.10 | 0.000187 |
| Zigong | 3185434 | 1353 | 0.0264 | 37.90 | 0.000425 |
| Panzhihua | 1083812 | 1319 | 0.0275 | 36.33 | 0.001217 |
| Luzhou | 4777796 | 1194 | 0.0274 | 36.51 | 0.000250 |
| Deyang | 3833494 | 1256 | 0.0267 | 37.42 | 0.000328 |
| Mianyang | 5316949 | 1607 | 0.0267 | 37.46 | 0.000302 |
| Guangyuan | 3037187 | 1270 | 0.0323 | 30.95 | 0.000418 |
| Suining | 3752672 | 1345 | 0.0254 | 39.44 | 0.000358 |
| Neijiang | 4211754 | 1385 | 0.0288 | 34.75 | 0.000329 |
| Leshan | 3483122 | 1586 | 0.0245 | 40.73 | 0.000455 |
| Nanchong | 7311281 | 1539 | 0.0279 | 35.81 | 0.000210 |
| Meishan | 3410974 | 1213 | 0.0286 | 35.01 | 0.000356 |
| Yibin | 5189956 | 1484 | 0.0281 | 35.61 | 0.000286 |
| Guangan | 4541444 | 1227 | 0.0259 | 38.59 | 0.000270 |
| Dazhou | 6415972 | 1850 | 0.0287 | 34.78 | 0.000288 |
| Yaan | 1545714 | 1330 | 0.0340 | 29.41 | 0.000860 |
| Bazhong | 3651140 | 1521 | 0.0344 | 29.09 | 0.000417 |
| Ziyang | 4908432 | 1279 | 0.0288 | 34.74 | 0.000261 |
| Abazangzuqiangzuzizhizhou | 849020 | 2204 | 0.0171 | 58.38 | 0.002596 |
| Ganzizangzuzizhizhou | 927678 | 2221 | 0.0131 | 76.25 | 0.002394 |
| Liangshanyizuzizhizhou | 4288625 | 2252 | 0.0232 | 43.06 | 0.000525 |
| Guiyang | 3460497 | 1479 | 0.0282 | 35.48 | 0.000427 |
| Liupanshui | 2824073 | 1575 | 0.0275 | 36.40 | 0.000558 |
| Zunyi | 7245127 | 1611 | 0.0269 | 37.19 | 0.000222 |
| Anshun | 2573257 | 1387 | 0.0311 | 32.19 | 0.000539 |
| Tongrendiqu | 3860920 | 1394 | 0.0326 | 30.71 | 0.000361 |
| Qianxinanbuyizumiaozuzizhizhou | 3083945 | 1476 | 0.0315 | 31.71 | 0.000479 |
| Bijiediqu | 6833368 | 1885 | 0.0298 | 33.57 | 0.000276 |
| Qiandongnanmiaozudongzuzizhizhou | 4259509 | 1480 | 0.0581 | 17.22 | 0.000347 |
| Qiannanbuyizumiaozuzizhizhou | 3830950 | 1465 | 0.0317 | 31.55 | 0.000382 |
| Kunming | 5053081 | 1951 | 0.0428 | 23.38 | 0.000386 |
| Qujing | 5880730 | 1783 | 0.0282 | 35.48 | 0.000303 |
| Yuxi | 2097531 | 1193 | 0.0402 | 24.85 | 0.000569 |
| Baoshan | 2429710 | 1679 | 0.0562 | 17.80 | 0.000691 |
| Zhaotong | 5280747 | 2185 | 0.0270 | 36.99 | 0.000414 |
| Lijiang | 1120828 | 1829 | 0.0433 | 23.11 | 0.001632 |
| Puer | 2379633 | 1679 | 0.0554 | 18.05 | 0.000706 |
| Lincang | 2188216 | 1898 | 0.0630 | 15.87 | 0.000867 |
| Chuxiongyizuzizhizhou | 2568224 | 1919 | 0.0459 | 21.76 | 0.000747 |
| Honghehanizuyizuzizhizhou | 4066600 | 1652 | 0.0517 | 19.36 | 0.000406 |
| Wenshanzhuangzumiaozuzizhizhou | 3403287 | 1716 | 0.0374 | 26.72 | 0.000504 |
| Xishuangbannadaizuzizhizhou | 881328 | 1936 | 0.0578 | 17.30 | 0.002197 |
| Dalibaizuzizhizhou | 3427088 | 2155 | 0.0550 | 18.19 | 0.000629 |
| Dehongdaizujingpozuzizhizhou | 1063986 | 1724 | 0.0251 | 39.83 | 0.001620 |
| Nujianglisuzuzizhizhou | 480838 | 1643 | 0.0439 | 22.77 | 0.003417 |
| Diqingzangzuzizhizhou | 340594 | 1857 | 0.0255 | 39.25 | 0.005452 |
| Lasa | 438549 | 1450 | 0.0273 | 36.67 | 0.003306 |
| Changdudiqu | 565602 | 1527 | 0.0248 | 40.30 | 0.002700 |
| Shannandiqu | 319560 | 989 | 0.0346 | 28.87 | 0.003095 |
| Rikazediqu | 662828 | 1249 | 0.0374 | 26.72 | 0.001884 |
| Naqudiqu | 394040 | 1583 | 0.0261 | 38.36 | 0.004017 |
| Alidiqu | 50758 | 709 | 0.0316 | 31.63 | 0.013968 |
| Linzhidiqu | 146839 | 1110 | 0.0244 | 41.05 | 0.007559 |
| Xian | 7488743 | 2074 | 0.0360 | 27.80 | 0.000277 |
| Tongchuan | 840467 | 993 | 0.0347 | 28.79 | 0.001181 |
| Baoji | 3694830 | 1525 | 0.0392 | 25.52 | 0.000413 |
| Xianyang | 4959532 | 1835 | 0.0364 | 27.49 | 0.000370 |
| Yanglingqu | 150033 | 734 | 0.0367 | 27.23 | 0.004892 |
| Weinan | 5355823 | 1857 | 0.0376 | 26.62 | 0.000347 |
| Yanan | 2087707 | 1145 | 0.0366 | 27.33 | 0.000548 |
| Hanzhong | 3768332 | 1521 | 0.0320 | 31.22 | 0.000404 |
| Yulin | 3357825 | 1113 | 0.0390 | 25.66 | 0.000331 |
| Ankang | 2954735 | 1336 | 0.0306 | 32.72 | 0.000452 |
| Shangluo | 2437599 | 1302 | 0.0323 | 30.91 | 0.000534 |
| Lanzhou | 3133560 | 1507 | 0.0333 | 30.07 | 0.000481 |
| Jiayuguan | 173207 | 728 | 0.0353 | 28.32 | 0.004203 |
| Jinchang | 440894 | 756 | 0.0391 | 25.59 | 0.001715 |
| Baiyin | 1735487 | 1150 | 0.0415 | 24.11 | 0.000663 |
| Tianshui | 3517068 | 1451 | 0.0392 | 25.51 | 0.000413 |
| Wuwei | 1867684 | 1244 | 0.0393 | 25.43 | 0.000666 |
| Zhangye | 1268690 | 1074 | 0.0362 | 27.59 | 0.000847 |
| Pingliang | 2174230 | 1164 | 0.0363 | 27.56 | 0.000535 |
| Jiuquan | 911713 | 1163 | 0.0345 | 28.99 | 0.001276 |
| Qingyang | 2512766 | 1320 | 0.0354 | 28.23 | 0.000525 |
| Dingxi | 2915865 | 1421 | 0.0340 | 29.45 | 0.000487 |
| Longnan | 2681899 | 1663 | 0.0410 | 24.39 | 0.000620 |
| Linxiahuizuzizhizhou | 1939903 | 1250 | 0.1943 | 5.15 | 0.000644 |
| Gannanzangzuzizhizhou | 688083 | 1521 | 0.0195 | 51.34 | 0.002210 |
| Xining | 1824671 | 1723 | 0.0381 | 26.25 | 0.000944 |
| Haidongdiqu | 1503218 | 1563 | 0.0532 | 18.78 | 0.001040 |
| Haibeizangzuzizhizhou | 262326 | 1219 | 0.0616 | 16.24 | 0.004647 |
| Huangnanzangzuzizhizhou | 217821 | 1196 | 0.0197 | 50.70 | 0.005491 |
| Hainanzangzuzizhizhou | 397547 | 1561 | 0.0148 | 67.51 | 0.003927 |
| Guoluozangzuzizhizhou | 145036 | 1559 | 0.0125 | 79.70 | 0.010749 |
| Yushuzangzuzizhizhou | 295337 | 1479 | 0.0246 | 40.70 | 0.005008 |
| Haiximengguzuzangzuzizhizhou | 361575 | 1444 | 0.0258 | 38.82 | 0.003994 |
| Yinchuan | 1399262 | 1243 | 0.0370 | 27.02 | 0.000888 |
| Shizuishan | 723446 | 942 | 0.0376 | 26.62 | 0.001302 |
| Wuzhong | 1226480 | 1169 | 0.0638 | 15.67 | 0.000953 |
| Guyuan | 1491423 | 1194 | 0.0599 | 16.68 | 0.000801 |
| Zhongwei | 1016058 | 1048 | 0.0436 | 22.92 | 0.001031 |
| Wulumuqi | 1902390 | 1829 | 0.0247 | 40.49 | 0.000961 |
| Kelamayi | 254281 | 1122 | 0.0212 | 47.14 | 0.004412 |
| Tulufandiqu | 567197 | 1163 | 0.0365 | 27.43 | 0.002050 |
| Hamidiqu | 506566 | 1382 | 0.0215 | 46.58 | 0.002728 |
| Changjihuizuzizhizhou | 1344180 | 1786 | 0.0263 | 37.96 | 0.001329 |
| Boertalamengguzizhizhou | 439337 | 1492 | 0.0190 | 52.73 | 0.003396 |
| Bayinguolengmengguzizhizhou | 1071685 | 1698 | 0.0187 | 53.56 | 0.001584 |
| Akesudiqu | 2043797 | 1565 | 0.0422 | 23.69 | 0.000766 |
| Kezilesukeerkezizizhizhou | 477688 | 1164 | 0.0498 | 20.07 | 0.002437 |
| Kashidiqu | 3642131 | 1786 | 0.0628 | 15.93 | 0.000490 |
| Hetiandiqu | 1806696 | 1168 | 0.0695 | 14.38 | 0.000646 |
| Yilihasakezizhizhou | 2506597 | 2522 | 0.0194 | 51.53 | 0.001006 |
| Tachengdiqu | 994368 | 1864 | 0.0194 | 51.50 | 0.001875 |
| Aletaidiqu | 623157 | 1548 | 0.0189 | 52.91 | 0.002484 |
| Shihezi | 600157 | 1180 | 0.0303 | 33.02 | 0.001966 |
| Alaer | 135565 | 765 | 0.0264 | 37.82 | 0.005643 |
| Wujiaqu | 33550 | 599 | 0.0315 | 31.74 | 0.017854 |
|  |  |  |  |  |  |
| **Mean** | 3528211 | 1313 | 0.0365 | 30.41 | 0.000905 |
| **Standard error** | 2904385 | 437 | 0.0149 | 9.45 | 0.001631 |
| **Minimum** | 33550 | 369 | 0.0126 | 5.15 | 0.000087 |
| **Maximum** | 30968906 | 3185 | 0.1943 | 79.70 | 0.017854 |


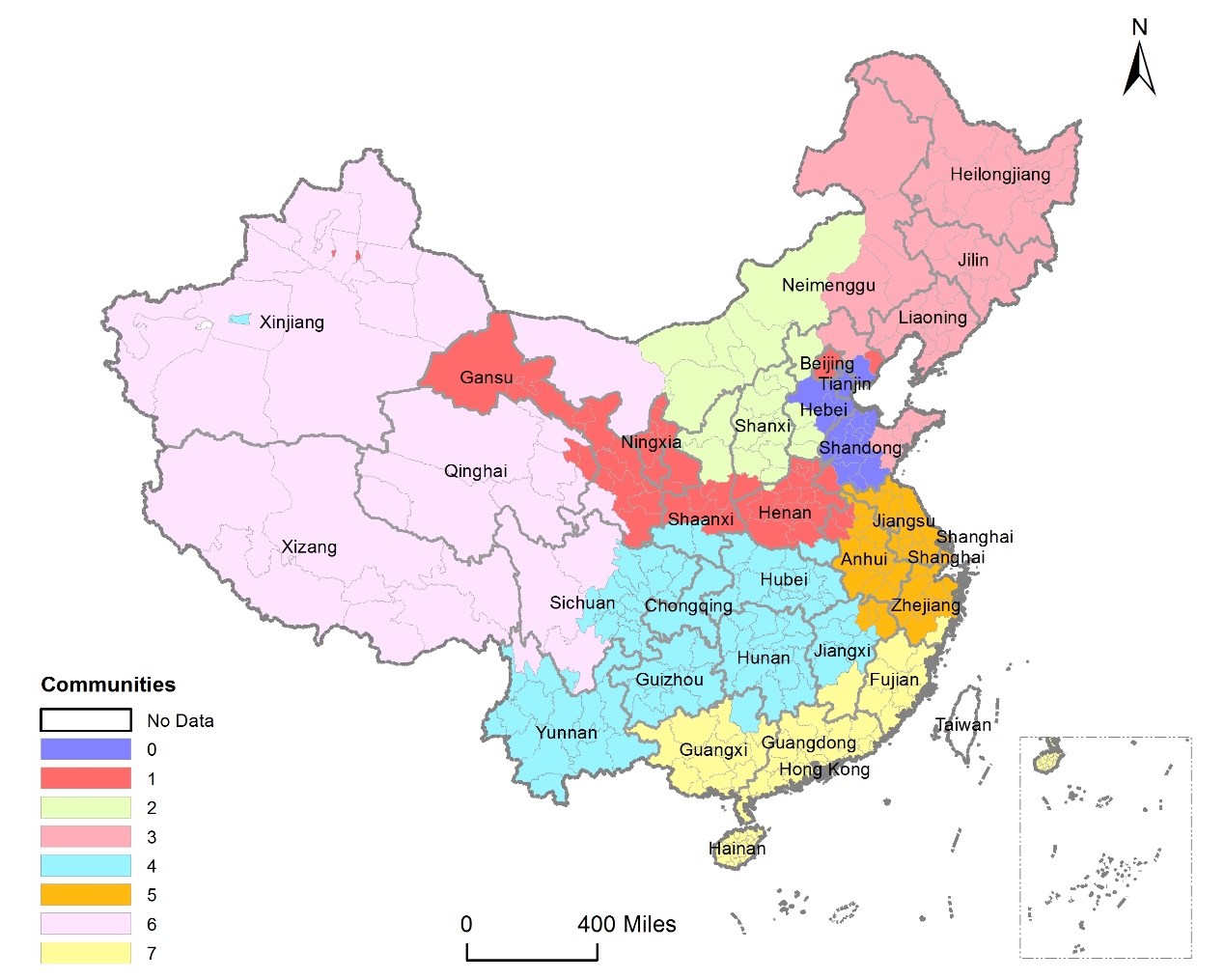


Figure S1 – Map showing the allocations of 8 communities in 9-layer MMST.


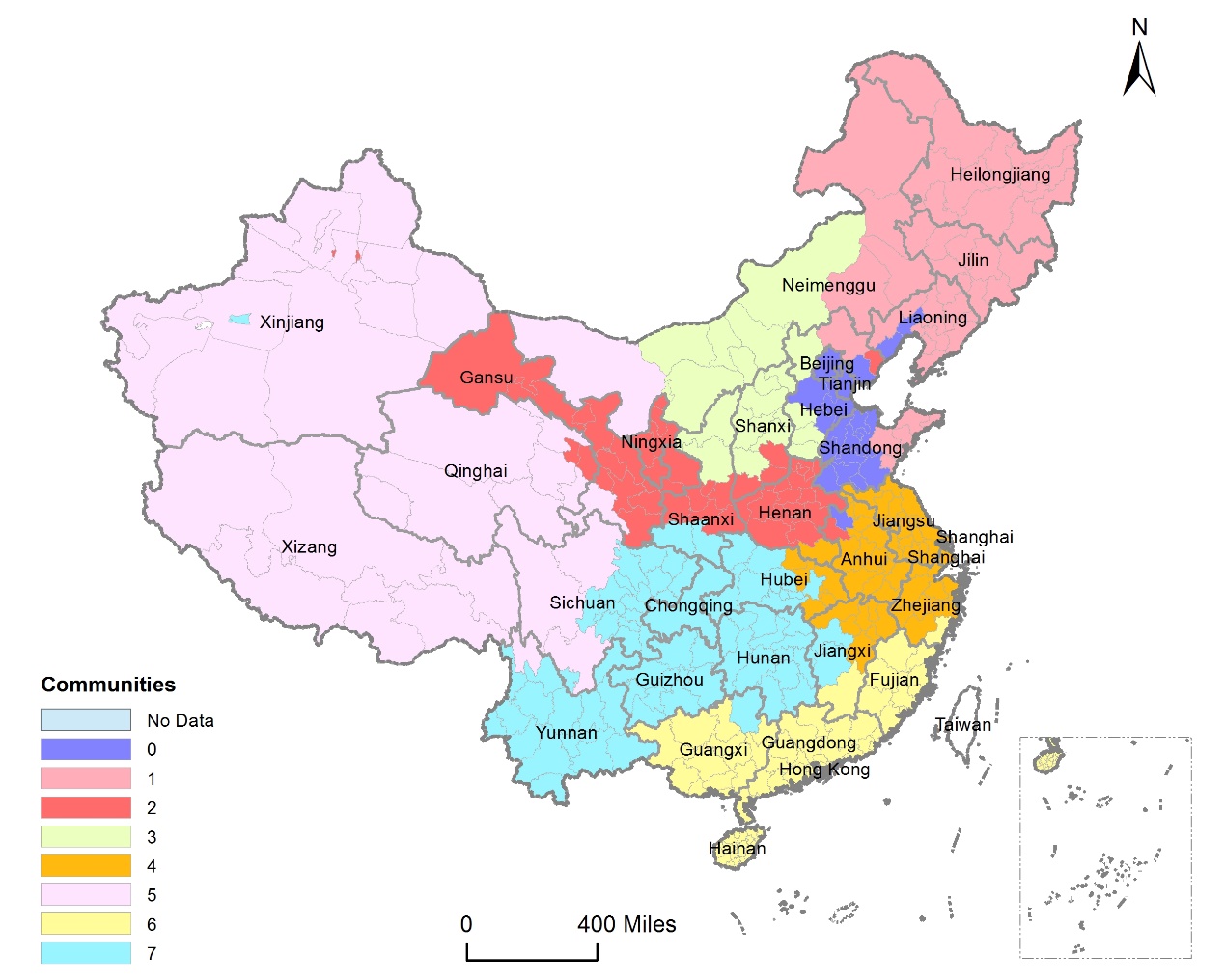


Figure S2 – Map showing the allocations of 8 communities in 11-layer MMST.


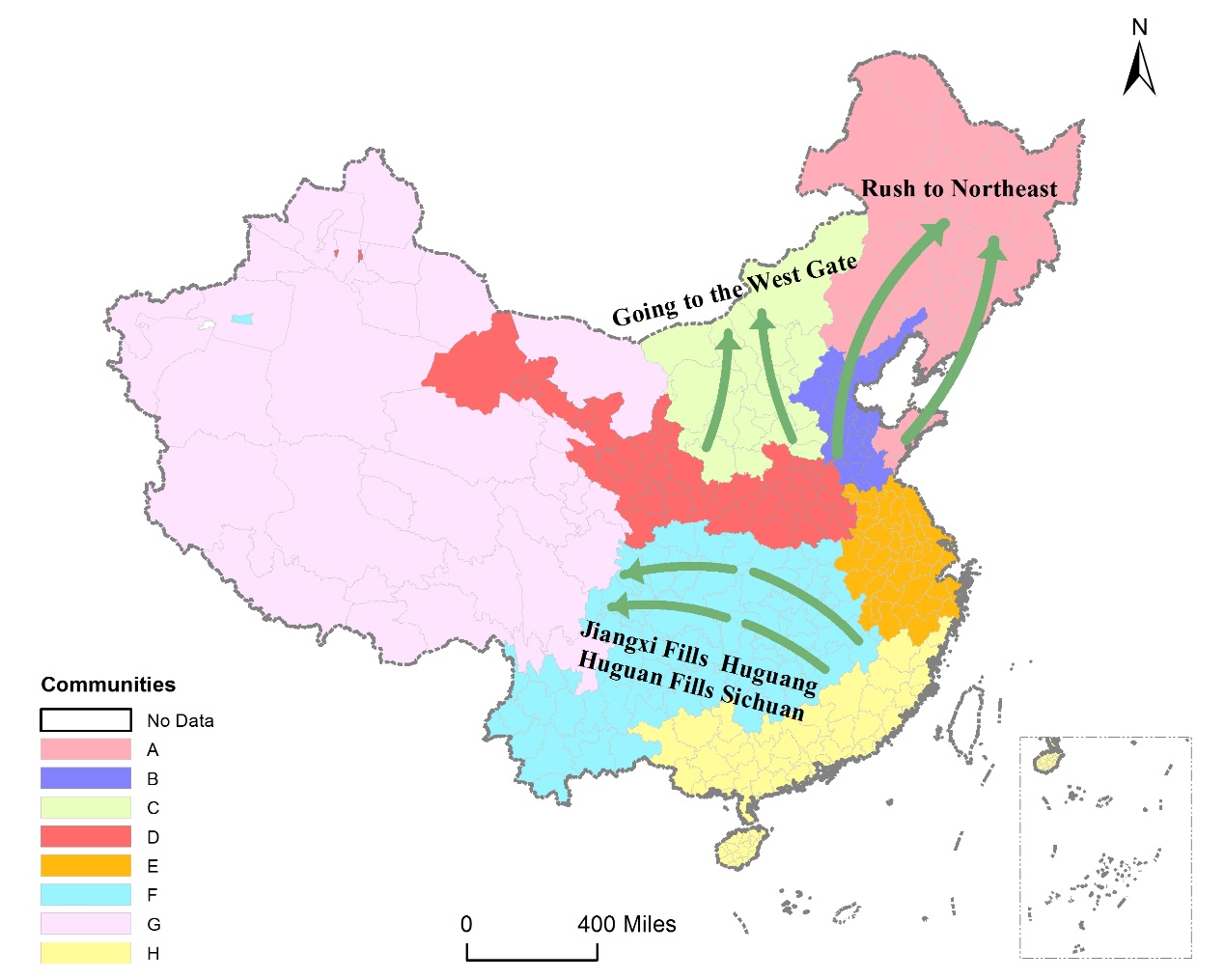


Figure S3 – Map showing the routes of three historical migrations: Rush to Northeast, Going to the West Gate, and Jiang Fills Huguang & Huguang Fills Sichuan.


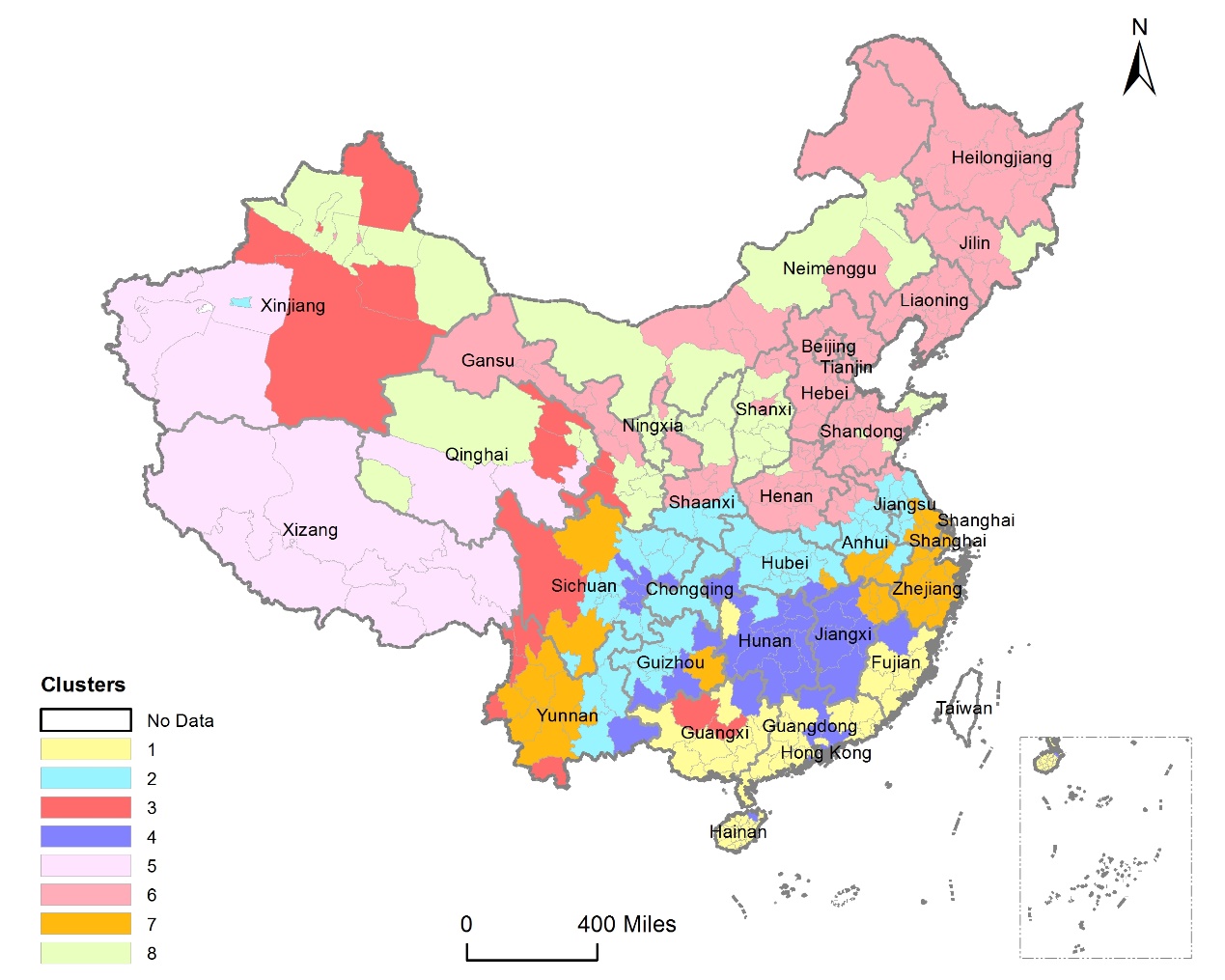


Figure S4 – Map showing the allocations of 8 clusters derived from K-means clustering.
